# Neighbourhood contacts and trade movements drive the regional spread of bovine viral diarrhoea virus (BVDV)

**DOI:** 10.1186/s13567-019-0647-x

**Published:** 2019-04-29

**Authors:** Luyuan Qi, Gaël Beaunée, Sandie Arnoux, Bhagat Lal Dutta, Alain Joly, Elisabeta Vergu, Pauline Ezanno

**Affiliations:** 1 BIOEPAR, Oniris, INRA, CS40706, 44307 Nantes, France; 2 MaIAGE, INRA, Université Paris-Saclay, 78350 Jouy-en-Josas, France; 3 Groupement de Défense Sanitaire de Bretagne, 56019 Vannes, France

## Abstract

To explore the regional spread of endemic pathogens, investigations are required both at within and between population levels. The bovine viral diarrhoea virus (BVDV) is such a pathogen, spreading among cattle herds mainly due to trade movements and neighbourhood contacts, and causing an endemic disease with economic consequences. To assess the contribution of both transmission routes on BVDV regional and local spread, we developed an original epidemiological model combining data-driven and mechanistic approaches, accounting for heterogeneous within-herd dynamics, animal movements and neighbourhood contacts. Extensive simulations were performed over 9 years in an endemic context in a French region with high cattle density. The most uncertain model parameters were calibrated on summary statistics of epidemiological data, highlighting that neighbourhood contacts and within-herd transmission should be high. We showed that neighbourhood contacts and trade movements complementarily contribute to BVDV spread on a regional scale in endemically infected and densely populated areas, leading to intense fade-out/colonization events: neighbourhood contacts generate the vast majority of outbreaks (72%) but mostly in low immunity herds and correlated to a rather short presence of persistently infected animals (P); trade movements generate fewer infections but could affect herds with higher immunity and generate a prolonged presence of P. Both movements and neighbourhood contacts should be considered when designing control or eradication strategies for densely populated region.

**Electronic supplementary material:**

The online version of this article (10.1186/s13567-019-0647-x) contains supplementary material, which is available to authorized users.

## Introduction

In managed metapopulations such as farming systems, endemic pathogens commonly spread at local and regional levels through different routes of transmission. Trade movements are primarily the consequence of constant renewal of the breeding stock to sustain productivity and herd size [1–3] and could happen between farms within either short or long distances, resulting in the possible introduction of infectious animals into the destination herds (e.g. [4–10]). Pathogens can also spread among farms within close proximity through direct contacts of animals or indirect contacts via agents (human, other animals, insects, and contaminated tools). Such a transmission route, named thereafter as neighbourhood contacts, is of particular importance in densely populated areas [11]. Other routes, such as environmental routes (air), also contribute to the spread of some pathogens [12, 13].

Assessing the contributions of different transmission mechanisms to pathogen spread provides policymakers with valuable information on how to allocate limited disease control resources more effectively over the course of a control program. In this context, modelling has been proven as an efficient approach to investigate the spread mechanisms of pathogens and to assess the impact of control strategies (e.g. [14] for bovine tuberculosis; [15] for bluetongue in cattle and sheep; [16] for bovine paratuberculosis). This approach is complementary to observations especially when sufficient biological information is not available or when field studies are difficult to perform. Well-developed models could assist the evaluation of control strategies before their implementation in the field, which supports authorities with decision making.

Bovine viral diarrhoea is an endemic disease distributed worldwide among industrial cattle herds [17]. Bovine viral diarrhoea virus (BVDV) is transmitted both vertically and horizontally, via direct contacts, bodily secretions, and contaminated fomites [18]. Animals exposed are either persistently (after vertical transmission) or transiently (after horizontal transmission) infected. Persistently infected animals are the most important source of virus, continuously shedding BVDV during their lifetime, while transiently infected animals shed fewer viruses during a limited infection period [17, 18]. BVDV infection could reduce reproductive performance and milk production, and increase occurrence of other diseases in cattle, leading to significant economic losses [19–23]. For example, economic impacts estimated based on experiences in several European dairy industries (Denmark, Norway, and UK) range from 10 to 40 M$ per million calvings with annual incidence rate of 20% to 40% [22]. Various control or even eradication schemes have been implemented by farmers’ organizations in many countries [24–29]. While these schemes have been very successful in some countries, eradication remains a difficult task in others [28, 30–32].

Several authors already focused on BVDV spread at a regional scale, using either a statistical or a mechanistic approach. Using statistical modelling, both neighbourhood contacts and trade movements were highlighted as contributing to virus spread at large scale with contrasted results in dairy vs. beef cattle herds [33–35]. Combining statistical analyses and mechanistic modelling, the impact of the dynamical trade network on BVDV spread was particularly investigated, pointing out the role of purchasing young animals [36]. It was shown that strategies regulating animal trade should target either high-movement farms [37] or specific productive systems [38] to achieve efficient control. However, the respective contribution of both routes to new herd infections was not assessed neither related to herd characteristics. Although both within and between-herd infection dynamics were explored accounting for neighbourhood contacts and animal movements [39], herd heterogeneity and actual trade movements were not incorporated in that study which considered a homogeneous and rather small metapopulation of herds, a situation in which trade movements were shown to largely overpass neighbourhood contacts in explaining BVDV spread and persistence. Very recently [40], available data from the Irish eradication programme were reviewed and the expected added-value of modifying the current control strategy was quantified using an expert system model derived from a model developed in [41]. The authors proposed an original model of BVDV spread at large scale, but did not focus on the investigation of virus propagation and persistence within and between populations, which thus remains quite poorly documented at such scales.

From the point of view of control and eradication, a transmission routes of underestimated contribution can undermine the efficacy of pre-planned control programs. In addition, it has been shown that herd structure, between age-group transmissions, the probability of birth and the presence of persistently infected animals could significantly influence the BVDV within-herd spread [42–44]. This is directly related to within-herd infection prevalence, thus to the likelihood that contacts with an infected herd generate new outbreaks. Hence, these herd-specific features might indirectly impact virus spread at a regional scale. Therefore, carrying out a more comprehensive study including trade movements and neighbourhood contacts as transmission routes, and accounting for herd heterogeneity is required to explore BVDV propagation mechanisms in a region, for which a complete multiscale mechanistic model is necessary.

Our objective was to assess the relative contribution of trade movements and neighbourhood contacts to BVDV spread both within and between dairy cattle herds in an endemic setting. Using a novel stochastic multiscale epidemiological model calibrated for Brittany (Western France), based on summarized epidemiological data and cattle detention data, we assessed herd infection probability and highlighted the characteristics of infected herds according to the transmission route involved.

## Materials and methods

**Figure 1:**
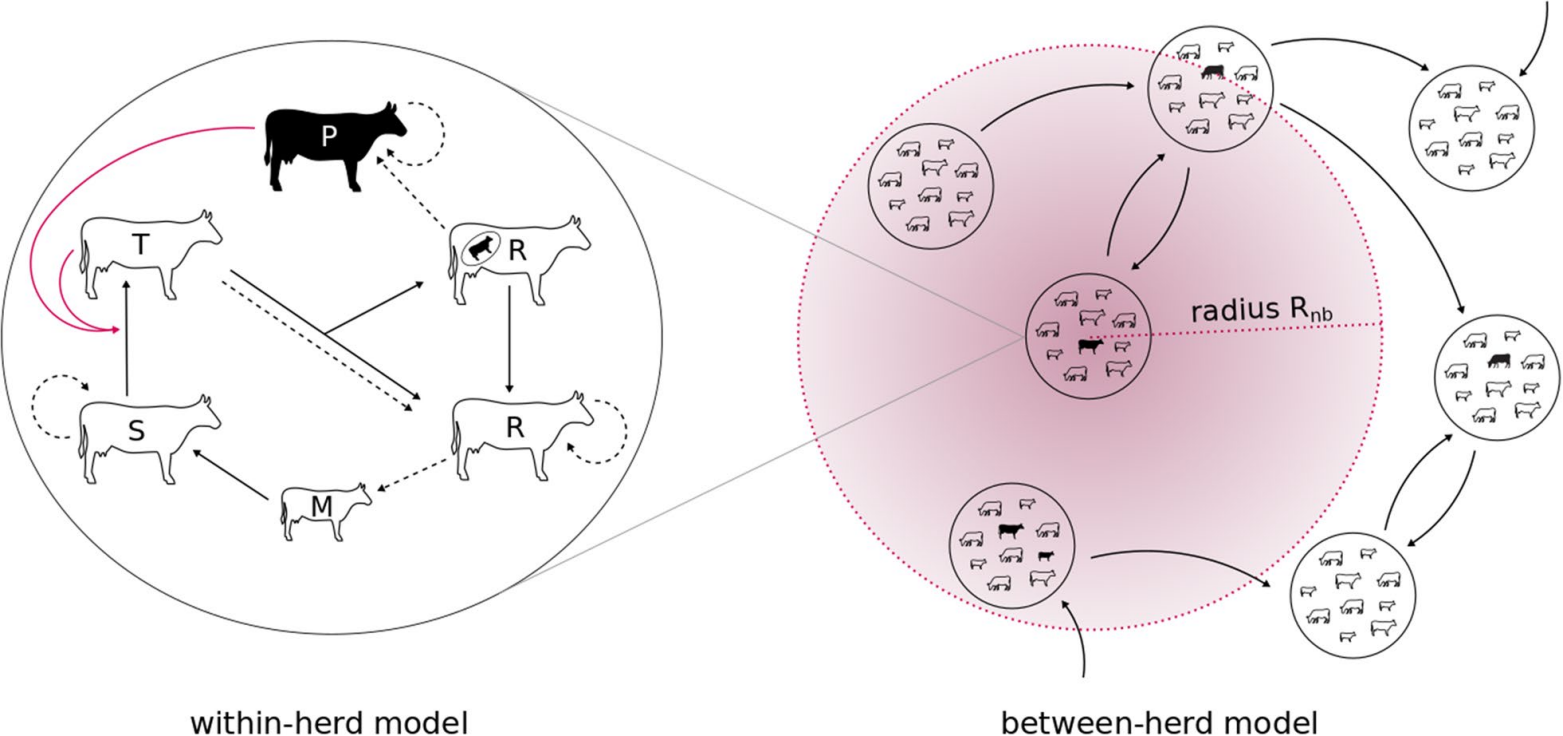
**General conceptual modelling framework of regional BVDV spread, coupling within- and between-herd infection dynamics.** On the left, the within herd dynamics with health states: S (susceptible), T (transiently infected), P (persistently infected), R (recovered, with the possibility of carrying a P foetus), M (protected by maternal antibodies). Dotted arrows represent birth, red arrows represent the influence on the force of infection, and black arrows correspond to transitions between states. On the right, the between-herd dynamics: herds are connected by directed trade movements (black arrows) and neighbourhood contacts (within radius R_nb_).

We developed a discrete time stochastic compartmental model describing within-herd demographics calibrated on herd-specific detention data and BVDV vertical and horizontal transmission dynamics. The within-herd modules are coupled at a regional between-herd scale through discrete trade movements represented explicitly from historical cattle movement data, as well as through neighbourhood contacts modelled as an infection pressure from infected herds located within a given radius around each exposed herd (Figure 1). The model was developed in C++.

**Figure 2:**
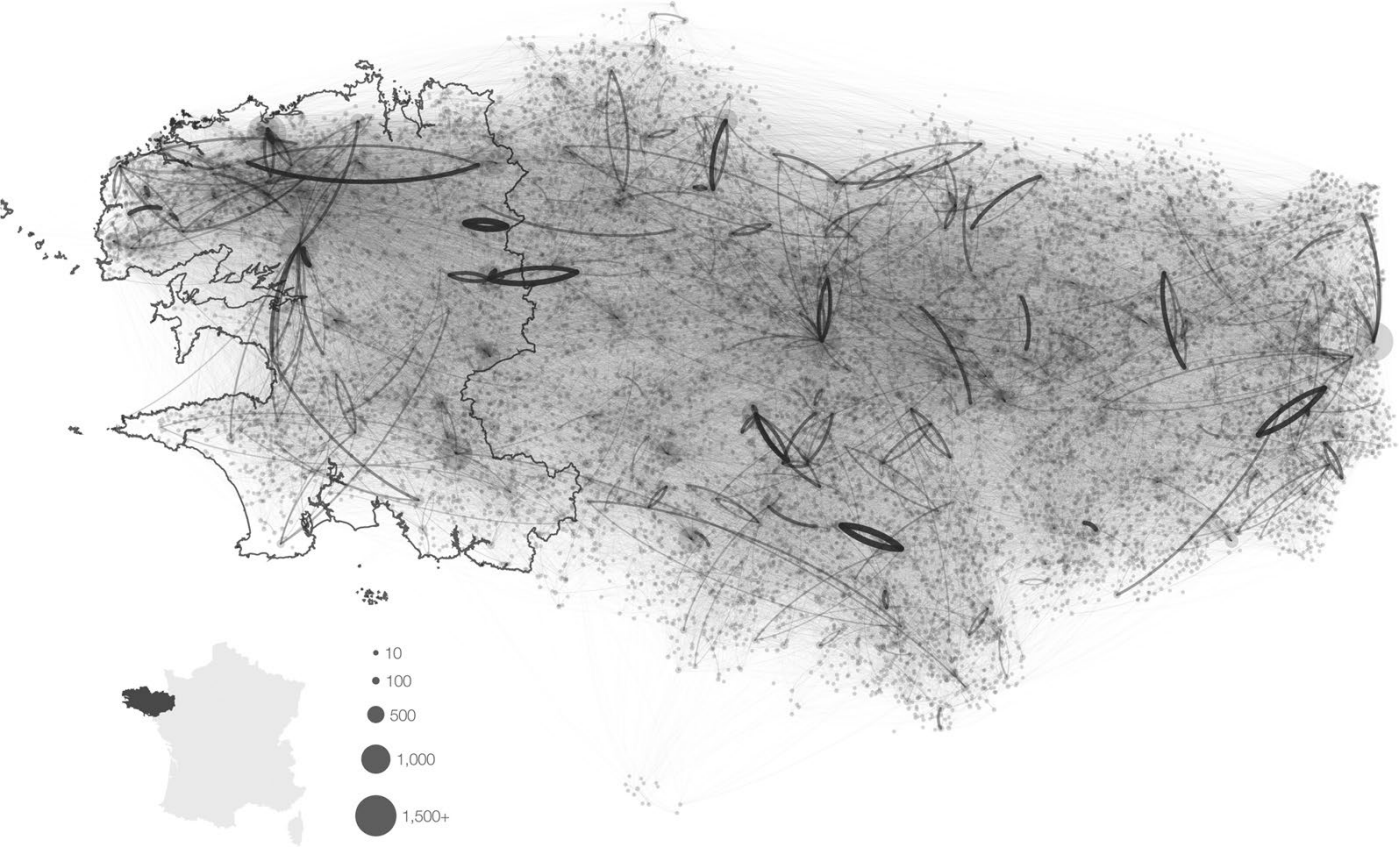
**Farm locations (randomly distributed within communes they belong to) and trade network (Brittany, France).** Each dot represents one farm included in the model (12 750 farms, belonging to 1173 communes). Each line corresponds to aggregated animal movements occurring between two connected farms over the period 2005–2013 (links are not directed for visual simplicity, but are in the model; line thickness is proportional to the number of traded animals). The western continuous border delimitates the Finistère department.

We modelled over the period 1/1/2005–31/12/2013 a metapopulation composed of 12 750 dairy cattle herds located in Brittany (Figure 2), a region in Western France densely populated with dairy herds mostly calving year-round using artificial insemination. Selected herds corresponded to all of the dairy herds located in Brittany and having more than 10 adult females and a sustained activity over the 9-year period. Since these herds are mainly composed of females, males were not kept in the model. In addition, summarized information from epidemiological data and the exact herd geographical coordinates were available for a subset of the metapopulation composed of 2652 dairy herds located in the Finistère department (i.e. 21% of the dairy herds from Brittany distributed over 20% of the area). This subset was used for qualitatively calibrating the model as regards the most unknown epidemiological parameters.

### Data

#### Animal detention and trade movements

The French National Cattle Database (FNCD) is a comprehensive database describing individual cattle life trajectories from birth to death. Cattle are defined by their national identification number, their breed and sex, and their date of birth, which enables all of the demographic parameters (birth rates and age-specific outgoing rates other than trade movements) to be estimated per herd per year (Table 1).

**Table 1:** **Default parameter values used in simulations**

Parameters and definitions	Values	References
$r_{h}^{g}$: herd- and age-specific outgoing rates (/2 weeks)	[0,1]	Calibrated on data
$r_{h}^{b}$: herd-specific birth rate (/year)	[0,0.36]	Calibrated on data
$s_{r}$: sex-ratio	0.5	Calibrated on data
$m_{P}$: rate of mortality of P (/2 weeks)	0.026	[19, 48]
$m_{P0}$: probability of mortality of P calves at birth	0.01	[19, 48]
$\beta_{w}^{P}$: within-group transmission rate for P (/day)	0.5	[49]
$\beta_{w}^{T}$: within-group transmission rate for T (/day)^a^	*0.03*, ***0.06***	[19]
$\beta_{b}^{P}$: between-group transmission rate for P (/day)^a^	*0.1*, ***0.2***	[50, 51]
$\beta_{nb}^{P}$: between-neighbour transmission rate for P (/day)^a^	*0.05*, 0.1, ***0.2***	Unknown
$R_{nb}^{{}}$: neighbourhood circle radius (km)^a^	*2*, ***4***, 6	Unknown

^a^These parameters were included in the sensitivity analysis. Values in italic are the initial default values, bolditalic values are the chosen values after qualitative comparison between simulated herd seroprevalence and incidence and field summarized epidemiological data (Finistère, Brittany, France).

In addition, the FNCD contains animal movements, with its date, the source and destination holdings (farms or commercial operators, i.e. markets and assembly centres, acting as intermediary nodes and as hubs of the trade network), as well as the causes and dates of entry into (birth, purchase) and exit from (death, sell) each holding. Pregnancy status was not available, neither the filial relationship between calves and dams. For a descriptive analysis of the national dataset, see [45, 46]. Only trade movements of females where the source or the destination holding belonged to the modelled metapopulation were accounted for. Movements to and from markets and assembling centres were not explicitly represented as the duration in these holdings is short (1 day in markets and ~3 days on average in assembly centres). Hence, movements going from farm A to a market or assembly centre and then to farm B were considered as direct movements from A to B in the model. A total of 1056 565 movements was considered, 226 553 occurring between farms of the metapopulation (Figure 2). The remaining movements concerned trade with partners located outside the metapopulation: 324 430 movements went into and 505 582 went out the metapopulation. The characteristics of the trade network in Finistère were closely in line with those of the whole metapopulation (Additional file 1A).

#### Herd locations

Herd locations were required to calculate distances between herds and to define neighbourhood contacts (see “Neighbourhood contacts” section). However, the exact locations were not known except for the Finistère subset for which GPS coordinates of the main farm building were available. Nevertheless, information in the FNCD enables to assign each herd uniquely to a commune (the smallest administrative division in France). Hence, we used the geo-coordinates of French commune borders to randomly distribute herds within their commune (R package {maptools}). The metapopulation area was composed of 1173 communes. The negligible impact of this procedure of geo-coordinates assignment was assessed by comparing model predictions obtained in Finistère when using the exact herd locations vs. the randomly assigned ones (Additional file 1B).

#### Summarized epidemiological data at a regional level

In the period 2000–2003, before the implementation of the regional control strategy for BVD in Brittany, the Animal Health Services (AHS) followed twice a year serological status of herds using ELISA tests on collected milk. Herds were considered positive if their seroprevalence was above 10% [26]. AHS provided summary statistics (ranges) on within-herd seroprevalence and herd incidence for herds located in Finistère. Seroprevalence in herds ranged from 54 to 62% during the endemic phase of BVDV spread (i.e. during the pre-control period), in agreement with previous observations made on a smaller subset of herds [26]. Yearly herd incidence ranged from 83 to 390 newly infected herds over this period (a herd being considered as newly infected if it was tested negative at two consecutive sample points during the year preceding two consecutive positive tests).

### Within-herd infection dynamics

The within-herd infection dynamics was adapted from a previously published model fully described in [42]. In brief, it was a stochastic model in discrete-time with a 2-week time-step. It described within-herd demographic and infection processes, accounting for both vertical and horizontal transmissions. Thereafter are described the main specificities of this within-herd model and updates implemented before its integration into a regional model.

Dairy cattle herds were managed by age groups, with more contacts within than between groups, thus inducing a heterogeneous within-herd contact structure. Four age-groups (calves, young heifers, heifers, and cows) and five health states (M: protected by maternal antibodies; S: susceptible; P: persistently infected; T: transiently infected; R: immune) were considered. Demography included birth, culling, and aging processes. Culling was performed before aging, using a Binominal distribution. The number of animals ($N_{h}^{X,g}$) in age group *g* and health state *X* in herd *h* was updated (Eq. 1) using *r*_*h*_^*g*^, the herd-specific outgoing probability in age group *g*.

1 $$N_{h}^{X,g} \left( {t + 1} \right)\sim Bin\left( {N_{h}^{X,g} \left( t \right),1 - r_{h}^{g} } \right)$$

Horizontal transmission gave rise to a transition from health state S to health state T based on age-specific transmission probabilities ($prob_{h}^{ inf,g}$, Eq. 2), accounting for transmission within and between age groups (Eq. 3) within the herd, as well as a between-herd transmission term due to neighbourhood contacts (Eq. 4):

2 $$prob_{h}^{ inf,g} \left( t \right) = 1 - e^{{ - \left( {f_{h}^{ inf,g} \left( t \right) + f_{h}^{ nb, inf,g} \left( t \right)} \right)}}$$

The within-herd force of infection $f_{h}^{ inf,g} \left( t \right)$ in age group *g* at time *t* assumed a frequency-dependent transmission [42, 44] (Eq. 3):

3 $$f_{h}^{ inf,g} \left( t \right) = \beta_{w}^{P} \frac{{N_{h}^{P,g} \left( t \right)}}{{N_{h}^{g} \left( t \right)}} + \beta_{w}^{T} \frac{{N_{h}^{T,g} \left( t \right)}}{{N_{h}^{g} \left( t \right)}} + \frac{{\beta_{h}^{P,g} }}{{N_{h}^{g} \left( t \right)}}\mathop \sum \limits_{a \ne g} \frac{{N_{h}^{P,a} \left( t \right)}}{{N_{h}^{a} \left( t \right)}}$$

where $N_{h}^{X,g} \left( t \right)$ was defined above and $N_{h}^{g} \left( t \right)$ was the total number of animals in age group *g* at time t, irrespective to the health state. Transmission rates $\beta$ are defined in Table 1. After infection, T animals recovered in one time-step [42], and became immune (R) for life [18].

The vertical transmission could lead to the birth of P calves if dams were infected in mid-pregnancy [47] or if they were P themselves. A birth event module (Additional file 1C) was developed to represent vertical transmission as no exact information was available on dam-calf pairs in data. At each time-step, it enabled to associate to each newborn his own health state (M, S, P, or R).

### Between-herd infection dynamics

#### Trade movements

Required information, such as age groups of moving animals, dates of exits and entries, source and destination herds, were taken directly from the FNCD. The missing information only concerned the health state of moving animals $(X_{source \to destination} )$, which was drawn using Multinomial distributions and probabilities $(prob_{source}^{{\left\{ {S,M,T,P,R} \right\},g}} )$ computed on simulated epidemic dynamics in the relevant age groups in source herds. If the animals at move came from outside the metapopulation, $prob_{source\;not\;in\;metapop}^{{\left\{ {S,M,T,P,R} \right\},g}}$ was assumed  equal to the simulated distributions among health states of all animals present in the metapopulation. This implicitly means that the metapopulation is a representative subset of a larger cattle population.

#### Neighbourhood contacts

As pastures of a given herd are not contiguous and can be located far from farm buildings and thus potentially be close to distant farms, the neighbourhood of one herd was defined by a disk of radius $R_{nb}$ centred at herd location. All the herds were assumed to have the same pasturing season, from mid-March to mid-November, which was relevant for Brittany region. During the pasturing season, animals belonging to the age groups of young heifers, heifers, and cows were assumed to be exposed to neighbourhood contacts. It was assumed that only P animals contributed to infection via neighbourhood contacts, as T animals only shed the virus for a short amount of time and at much lower levels than P animals [19]. A herd was assumed to be equally exposed to all P pasturing animals from any herd located in its neighbourhood. The force of infection due to neighbourhood contacts ($f_{h}^{ nb, inf,g} \left( t \right)$, Eq. 4) in age group *g* in herd *h* at time *t* was modelled as:

4 $$f_{h}^{ nb, inf,g} \left( t \right) = \frac{{\beta_{nb}^{P} }}{{N_{h}^{g} \left( t \right)}}\frac{{N_{h}^{nb, P} \left( t \right)}}{{N_{h}^{nb} \left( t \right)}}$$

where $\beta_{nb}^{P}$ was the between-neighbour transmission rate due to P pasturing animals (Table 1), $N_{h}^{g} \left( t \right)$ was defined above, $N_{h}^{nb, P} \left( t \right)$ and $N_{h}^{nb} \left( t \right)$ were the number of P animals and the total number of animals, respectively, on pastures in the neighbourhood of herd *h* at time *t*.

### Model outputs

Model outputs were generated for two distinct and complementary purposes: first, model calibration based on a comparison with summarized epidemiological data and on a sensitivity analysis; and second, scenario analysis to assess the contribution of the two transmission routes to BVDV spread and identify the drivers of infection through each of these two routes.

For model calibration, two outputs were recorded: (i) the herd seroprevalence, calculated as the proportion of herds with more than 10% of R animals, and (ii) the yearly test-based herd incidence, defined as the annual number of herds detected as newly infected. In both cases, the outputs were defined similarly as in epidemiological data to enable comparison. For herd incidence, a regular sampling scheme was implemented in the model, using the proportion of animals in health state R as a proxy for test result and with tests implemented every 6 months. Test result was assumed to be negative for herds having less than 10% of R animals, positive otherwise. A simulated herd was considered as detected as newly infected if it was negatively tested at two consecutive sampling points immediately before two consecutive positive tests. The number of newly detected herds was aggregated per year.

To assess the contribution of the two transmission routes to BVDV regional spread, six model outputs were generated. At each time step over 9 years, we recorded: (i) the number of infected herds in the metapopulation (i.e. herds with at least 1 P or T animal); (ii) the number of newly infected herds (i.e. herds with no P nor T animal at time *t* − 1 and at least 1 P or T animal at time *t*); (iii) the annual probability of a herd to be infected (*Prob_being_inf*), calculated as the proportion of runs in which the herd was infected over a year; (iv) the annual probability of a herd to be newly infected (*Prob_getting_inf*), calculated as the proportion of runs in which the herd was newly infected that year. As spontaneous fade-out is frequent for this disease, we also assessed (v) the herd re-infection probability, defined as the probability of a herd being newly infected at least twice over the 9-year period, and (vi) the proportion of presence time of at least one P animal over the 9 years in a specific herd.

### Simulation settings

For each herd, the initial herd structure was based on the corresponding demographic structure on 1/1/2005 as observed in the FNCD. To mimic a BVDV regional endemic situation, we first generated 100 runs over the 9 years of available trade data starting with 1 P cow randomly introduced into 10% randomly selected herds at 1/1/2005. All of the remaining animals were assumed to be susceptible. These preliminary simulations led to the generation of 100 infection dynamics gathering information for each herd. Then, this information was used as new starting conditions (i.e. the empiric distribution of initial conditions in endemic situations).

At each time step, herd specific state variables were updated thanks to three processes: aging, infection, and trade movements.

Parameters values used in simulations are given in Table 1. The number of runs, different depending on the analysis performed, is specified hereafter, when relevant.

### Sensitivity analysis and parameter calibration

A sensitivity analysis was performed using a complete factorial design on four parameters (40 scenarios): (i) the within-herd transmission rates of T animals within age groups, $\beta_{w}^{T}$ and (ii) of P animals between age groups, $\beta_{b}^{P}$, (iii) the between neighbouring herds transmission rate, $\beta_{nb}^{P}$, and (iv) the radius of the neighbourhood, $R_{nb}$ (Table 1). Tested values for $R_{nb}$ corresponded to an average number of neighbours of 6 (25^th^–75^th^ percentiles = 3–8, median = 6), 26 (25^th^–75^th^ percentiles = 18–32, median = 25), and 58 (25^th^–75^th^ percentiles = 44–70, median = 56), respectively for 2, 4 and 6 km radius. Model outputs analysed were the mean herd seroprevalence, the mean proportion of infected herds, and the mean yearly test-based herd incidence over time. Sensitivity indices for the dynamic outputs, either at each time step or on their summaries over the whole trajectory, were calculated using the R package {multisensi} (Additional file 1D). Then, simulated scenarios were compared with summarized field epidemiological data using two outputs: the average herd seroprevalence and the yearly test-based herd incidence. This comparison enabled the calibration of the model by selecting, among tested parameter values, the most relevant for subsequent analyses. This analysis was restricted to Finistère, a subset of the metapopulation for which summary statistics from data were available. A 6-year period was considered to limit simulation time. Fifty stochastic runs were performed for each of the 40 scenarios of the factorial design, which was sufficient to assess the variability of the selected averaged time-varying outputs.

### Scenario definition and analyses

First, three scenarios were compared (using the calibrated model) at the scale of Brittany: (1) transmission was possible by neighbourhood contacts only; (2) transmission was possible by animal trade movements only; (3) both transmission routes were possible. For each scenario, we looked at the number of infected and of newly infected herds over time. We carried out 100 runs for each scenario.

For the most complete scenario with both transmission routes, we also assessed whether herds at extreme percentiles (5 and 95) of the distribution of the probability of being infected (*Prob_being_inf*) could be discriminated by their number of neighbours and of animals purchased, and if these herds had a specific spatial distribution in Brittany (spatial clustering analysis using DBSCAN method). Then, we characterized the annual probability of getting infected (*Prob_getting_inf*) per causal transmission route using the Random Forest method (R package {randomForest}). Potential explanatory variables were per herd: the number of herds in the neighbourhood (nNB), the annual number of animals purchased (InStrength), the annual number of animals younger than 1 year of age purchased by the herd (InStrength_Less1yr), the annual number of source herds (InDegree), the annual number of animals purchased by neighbouring herds (InStrengthNB), the annual number of animals younger than 1 year of age purchased by neighbouring herds (InStrengthNB_less1yr), the proportion of cows aged 24 to 36 months (%24_36) in the herd, the number of females younger than 1 year of age (Size1yr) and the herd size (SizeTot). Furthermore, assuming herd re-infection probability was barely correlated to its probability to be infected (*Prob_being_inf*), we investigated the four possible patterns of successive infections: both by neighbourhood contacts, first by neighbourhood contacts and second by trade movements, both by trade movements, and first by trade movements and second by neighbourhood contacts. Analysing these pairs enabled to assess if herds were randomly infected with respect to the transmission route, or if they were more exposed to one or the other route (Additional file 1E).

Finally, we analysed how the within-herd infection dynamics and the causal herd infection route interacted. Limiting the analysis to herd-runs for which new infections occurred, the proportion of time over the 9 years a herd possessed at least one persistently infected animal was related to the proportion of these new infections which were due to neighbourhood contacts. The proportion of recovered animals in the herd at the start of a new infection was used as a proxy for herd immunity and its distributions were compared between causal infection routes.

## Results

### Sensitivity analysis and model calibration

**Figure 3:**
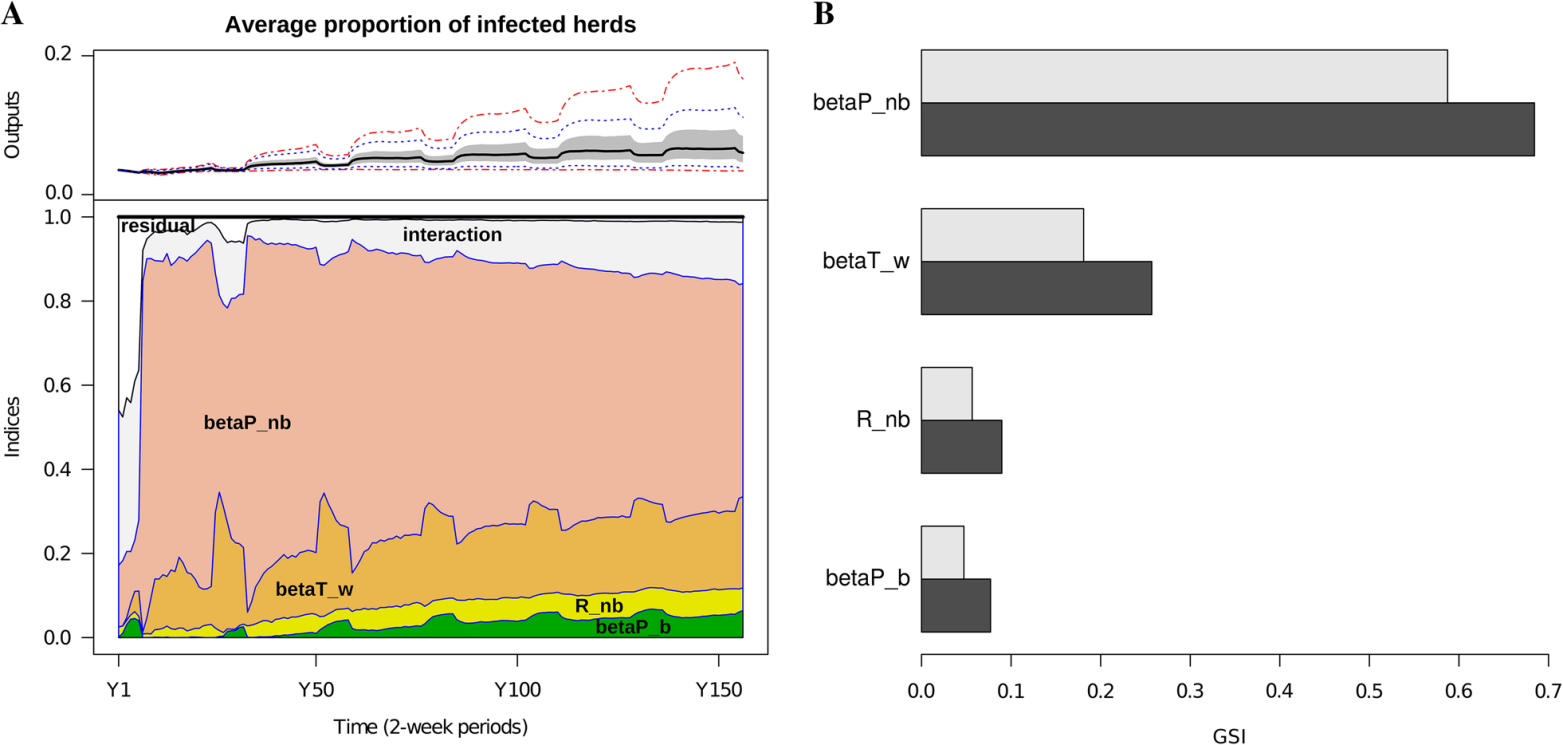
**Sensitivity analysis of the mean (over 50 runs) proportion of infected herds on 40 scenarios.** ANOVA was performed for **A** output values at each time-step, **B** the first component (inertia: 99%) of a PCA over all the time-point values (Additional file 1D). **A** Total sensitivities (as areas under the curve of the normalized variance) over time for tested parameters: betaP_nb corresponds to $\beta_{nb}^{P}$, betaT_w to $\beta_{w}^{T}$, R_nb to $R_{nb}^{{}}$, and betaP_b to $\beta_{b}^{P}$ (Table 1). **B** Global sensitivity indices (GSI) of the four parameters ranked in descending order. Values of sensitivity indices were split in main effect (white) and global effect (sum of main effect and interactions, black).

The predicted average proportion of infected herds (Figure 3) was highly sensitive to the transmission rate due to P animals in the neighbourhood ($\beta_{nb}^{P}$) (same behaviour for herd seroprevalence and herd incidence, data not shown). Indeed, this parameter had the largest sensitivity index both for the main and global effects, for all time-points of the outputs (Figure 3A). The second most influent parameter was surprisingly the transmission rate due to T animals within age-group ($\beta_{w}^{T}$), with one third of the global sensitivity due to interaction terms (Figure 3B), its effect being mainly potentiated by $\beta_{nb}^{P}$ (Additional file 1F, Figures S5 and S6). The sensitivity to variations in the neighbourhood radius $R_{nb}$, relatively small compared to the two first parameters, depended (positive correlation) on $\beta_{nb}^{P}$ (Additional file 1F, Figures S5 and S6). The impact of $R_{nb}$ was much less visible when varying it from 4 to 6 km, than from 2 to 4 km (Figure 3), due to a potential saturation effect since the number of neighbours was already large for $R_{nb} = 4km$ (50% of herds had more than 25 neighbours within this distance). The interactions of $R_{nb}$ with the other parameters accounted for one third of its global influence on the average proportion of infected herds (Figure 3B).

Based on visual comparison of model outputs (Additional file 1F, Figures S5 and S6) and summarized epidemiological data at the regional level, the most uncertain parameters were calibrated and set to the following values for subsequent simulations: $\beta_{nb}^{P}$ = 0.20, *R*_*nb*_= 4, $\beta_{w}^{T}$ = 0.06, $\beta_{b}^{P}$ = 0.20, other parameters being set at their reference value (Table 1). Using these parameter values, the predicted herd seroprevalence was 35% after 6 years when starting at 17% (Additional file 1F, Figure S5), which was lower than observations ranging from 54 to 62%. The predicted yearly test-based herd incidence was of 150 herds (Additional file 1F, Figure S6), in perfect agreement with observations ranging from 83 to 390 herds detected annually as newly infected.

### Contribution of neighbourhood contacts and trade movements to regional BVDV spread

**Figure 4:**
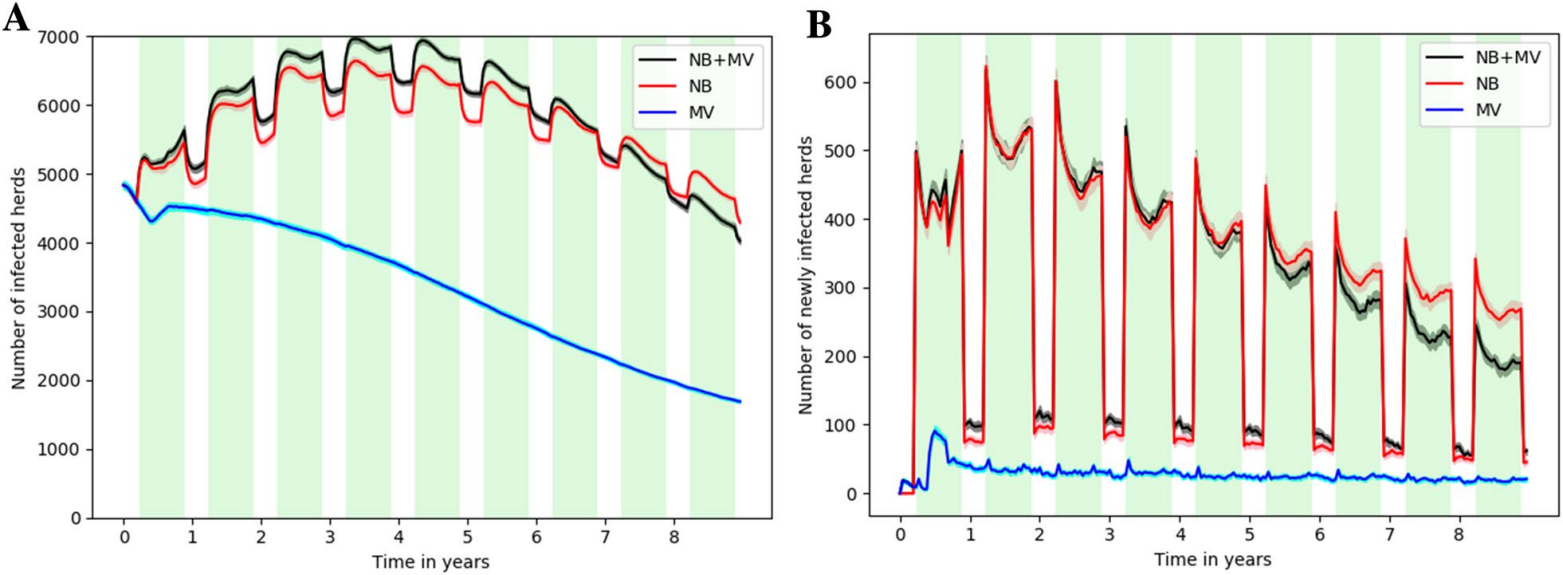
**Contribution of trade movements (MV) and neighbourhood contacts (NB) to regional BVDV spread. A** Number of infected herds (i.e. having at least one persistently (P) or transiently (T) infected animal) according to the transmission route activated (black: both routes (NB + MV); red: only NB; blue: only MV). **B** Number of herds getting newly infected (i.e. having one P or T animal at time *t* while none at time *t* − 1) when both transmission routes were activated, according to the causal route (red: NB; blue: MV). Green stripes show pasture periods during which neighbourhood contacts occurred, shadows show 25^th^–75^th^ percentiles illustrating stochastic fluctuations (100 runs), and lines correspond to median values.

BVDV persisted up to the end of the 9 years in all runs for the three scenarios. With both transmission routes (neighbourhood contacts and trade movements), the herd prevalence reached more than half of the 12 750 simulated herds (Figure 4A, black line). The number of infected herds showed a non-linear pattern when considering neighbourhood contacts only and when considering both routes, while it was mostly decreasing when considering trade movements only. This highlighted the crucial role played by neighbourhood contacts (Figure 4A). The occurrence of newly infected herds every 2 weeks, although decreasing, indicated a large spread throughout the metapopulation (Figure 4B). Transmission due to neighbourhood contacts gave rise to periodic variations in herd prevalence and incidence, with higher values during the pasture season. When only trade movements were considered, a much lower number of newly infected herds was predicted, but this incidence was almost consistent over the 9-year period, while the intensity of seasonal peaks decreased when considering neighbourhood contacts only. When both transmission routes were activated, 93.1% of new infections were due to neighbourhood contacts, 6.7% to trade movements, and 0.2% simultaneously to both routes.

### Herd infection: probabilities of contamination, persistence, and reinfection

**Figure 5:**
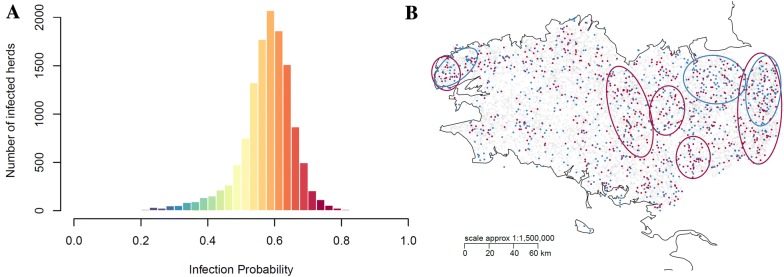
**Spatial analyses and empirical distribution of the annual probability of herd infection (i.e. to have at least one persistently or transiently infected animal once over the year). A** Histogram of the probability (*Prob_being_inf*), **B** spatial distribution of herds according to their probability (blue: herds with values lower than the 5^th^ percentile (0.42); purple: herds with values higher than the 95^th^ percentile (0.69); grey: other herds). Analyses were based on 100 runs.

Ninety percent of the simulated herds had quite a high annual probability to have a detected infected status (*Prob_being_inf* of 0.42 and 0.69 corresponding to the 5^th^ and 95^th^ percentiles, respectively; Figure 5A). Herds in extreme percentiles of this probability (< 0.42 vs. > 0.69) exhibited an intricate spatial pattern (Figure 5B). The geographical distribution of herds in these two populations was not discriminant, both populations being situated mainly in high-density areas of farms. Herds with a moderate to low risk (percentile 5) purchased similar number of animals but had significantly fewer neighbours than herds with the highest risk (percentile 95), and their neighbours purchased significantly fewer animals than neighbours of herds with the highest risk (percentile 95) (Additional file 1G, Figure S7).

**Figure 6:**
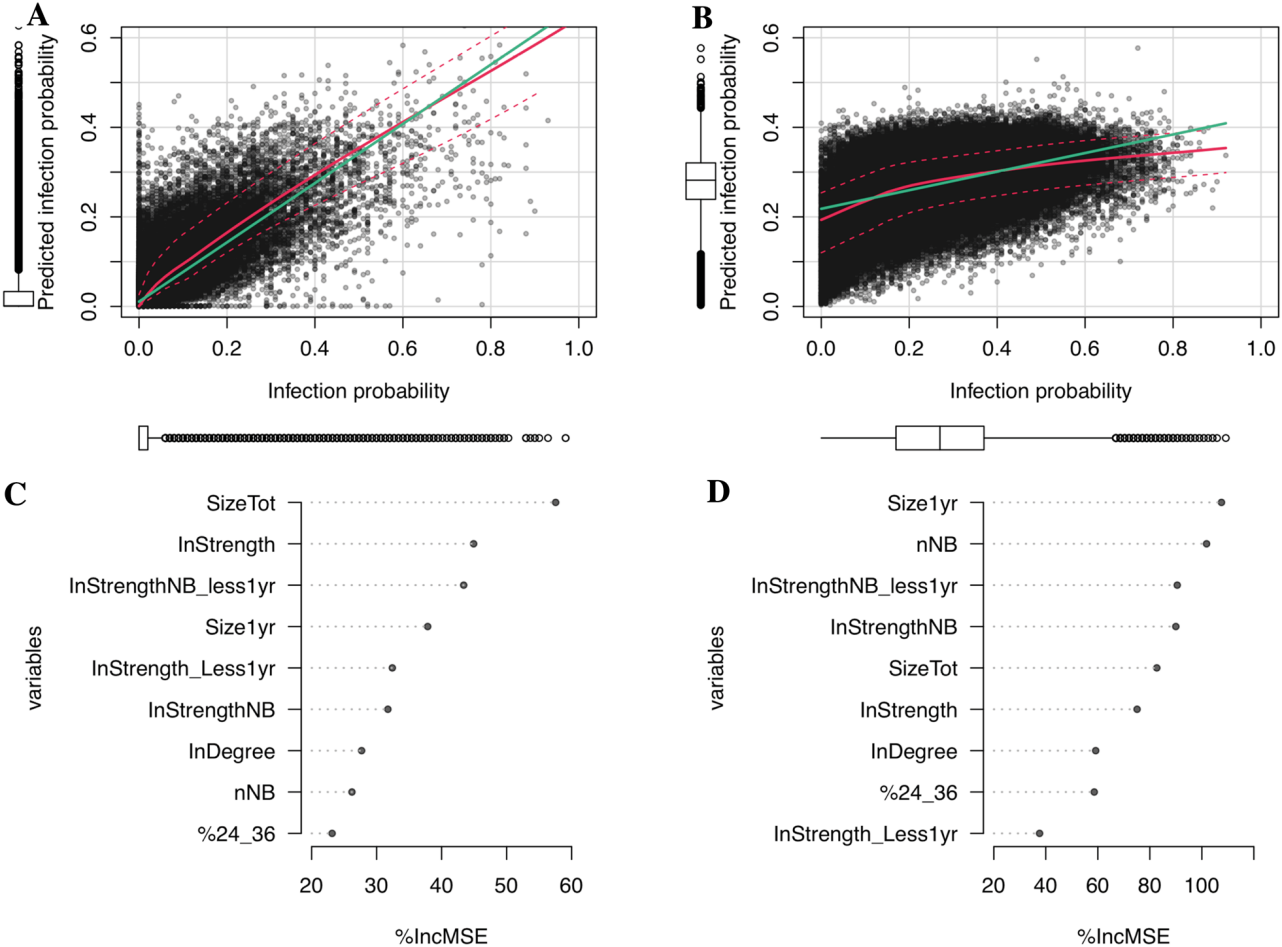
**Drivers of the annual probability of acquiring infection per causal transmission route (A and C trade movements, B and D neighbourhood contacts).** Newly infected herds had at least one transiently (T) or persistently (P) infected animal over the year while none the previous year (*Prob_getting_inf*). **A**, **B** Simulated vs. predicted probability by the Random Forest regression. Each point represents one herd-year. The green and red solid lines are respectively the regression line and the nonparametric-regression smooth. The red dotted lines are the estimations of the variance function. **C**, **D** Explanatory variables ranked by importance as measured by the Random Forest method (using %IncMSE, Additional file 1H). Variables with larger values are more important. Variables are: number of herds in the neighbourhood (nNB), annual number of animals purchased (InStrength), annual number of animals of less than 1 year purchased (InStrength_Less1yr), annual number of source herds (InDegree), annual number of animals purchased by neighbouring herds (InStrengthNB), annual number of animals of less than 1 year purchased by neighbouring herds (InStrengthNB_less1yr), proportion of cows aged 24–36 months (%24_36), herd size (SizeTot), number of females younger than 1 year (Size1yr). Analyses were based on 100 runs.

Explanatory variables selected using regression based on random forest explained 66% of the variability of the probability of new herd infections due to trade movements, and 20% of the variability of probability of new herd infections due to neighbourhood contacts (Figure 6). The three main explanatory variables of the probability of new herd infection due to movements were not correlated. Interestingly, one variable was a herd characteristics (size), one was related to movements (number of purchased animals), and one was related to both neighbourhood and movements (number of young animals purchased by neighbouring herds). Given the low proportion of explained variance, the model of the probability of new herd infection due to neighbourhood contacts was less relevant.

**Figure 7:**
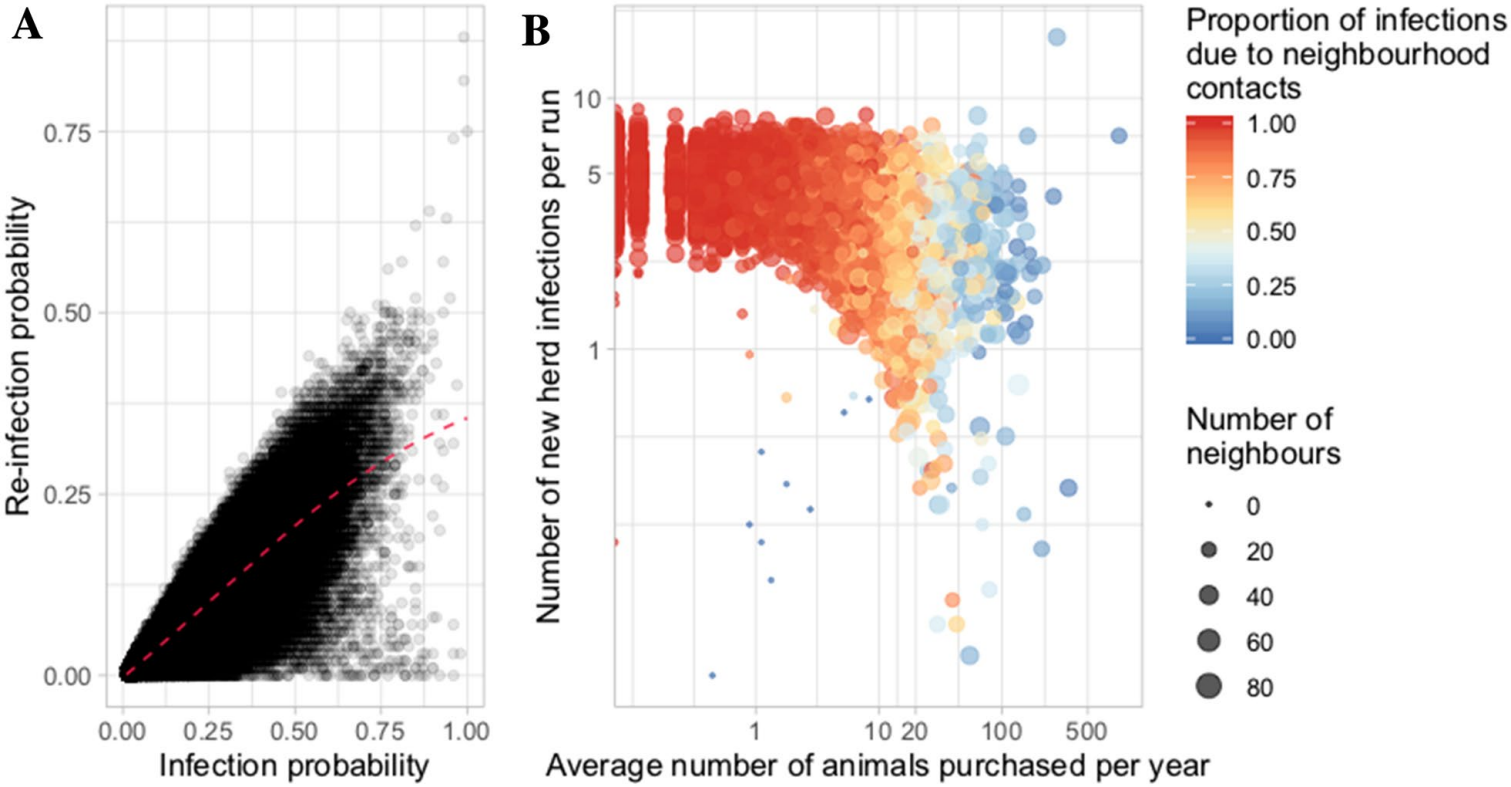
**Analysis of BVDV outbreaks at herd scale. A Re-infection probability vs. infection probability.** Each point represents one herd. The red line is the smoothed non-parametric regression line. **B** Number of new infections per herd-run, function of the average number of animals purchased per herd-year. The circle size is proportional to the number of neighbours and the colour is related to the proportion of new infections due to a specific transmission route (from red when only due to neighbourhood contacts to blue when only due to animal trade). Analyses were based on 100 runs.

In the metapopulation, 98% of the herds reported a positive re-infection probability. However, this re-infection probability was low (< 0.22) for 90% of them. The re-infection probability grew linearly with respect to the infection probability, with a slight saturating effect (Figure 7A). Among all herds and runs, 80% of herd-runs reported at least one BVDV outbreak, 72% of herd-runs were infected at least once by neighbourhood contacts, and 21% were infected at least once by trade movements. An increase in the mean number of purchased animals per year lowered the proportion of new herd infections due to neighbourhood contacts, while increasing the variability in the number of new infections per herd per run for a similar range of number of neighbours (Figure 7B). Among the 398 herds more prone to be infected through animal movements than through neighbourhood contacts, 96% purchased at least 10 animals per year. This highlighted a change in the main between-herd transmission route with purchase intensity.

The causal routes of two successive within-herd infections were not associated at random, with higher probability of having similar causal routes than expected, i.e. to be more exposed to one or the other route throughout time. Indeed, when the previous within-herd infection was caused by neighbourhood contacts (trade movements, respectively), the latter one was caused by the same route with probability 0.96 (0.47, respectively), whereas the probability for a herd to be infected by neighbourhood contacts (trade movements, respectively) was 0.93 (0.07, respectively). Herds only infected by neighbourhood contacts over all runs (17% among all herds) had on average 27 neighbours (range 1–65, median = 26) and purchased 0.1 animals per year (range 0–15, median = 0, percentile 95% = 4). Only 10 herds were infected exclusively by trade movements and they were all but one isolated herds.

### Interactions between within-herd dynamics and causal infection route

**Figure 8:**
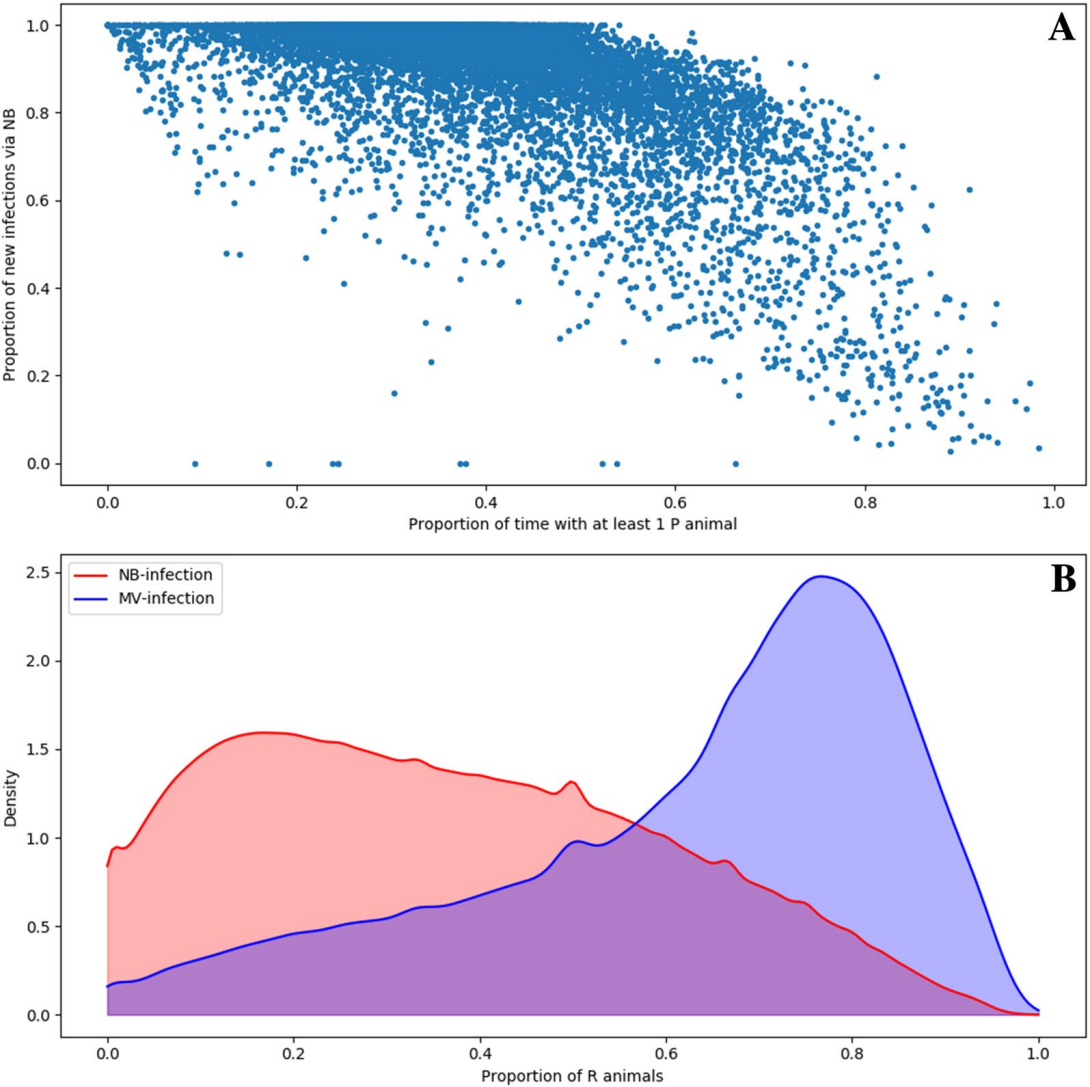
**Characteristics of the within-herd infection dynamics. A** Proportion of herd infections due to neighbourhood contacts with respect to the proportion of time (over 9 years) one persistently infected (P) animal was present in the herd. **B** Smoothed distribution of the proportion of immune (R) animals at the onset of infection per causal transmission route (red: neighbourhood contacts, blue: trade movements).

Most herds predominantly infected by neighbourhood contacts had at least one persistently infected animal less than half the simulation period (Figure 8A). However, the probability of having persistently infected animals in the herd during more than 75% of the time was still positive, even for herds only incidentally infected by neighbourhood contacts (e.g. less than 20% of new infections). The proportion of immune animals at the infection onset was significantly different in herds infected through neighbourhood contacts (most being less than 0.2) compared to herds infected by trade movements (most being close to 0.75; Figure 8B).

## Discussion

Predictions from a new multi-scale epidemiological model highlighted that neighbourhood contacts and trade movements complementarily contribute to BVDV spread on a regional scale in endemically infected and densely populated areas, leading to intense fade-out/colonization events: neighbourhood contacts generate the vast majority of outbreaks (72%) but mostly in low immunity herds and cause a rather short presence of persistently infected animals (P); trade movements generate fewer infections but could affect herds with higher immunity and generate a prolonged presence of P (Figure 8A). Infections caused by neighbourhood contacts predominantly generate transiently infected animals who recover in 2 weeks [17, 23]. While the impact of neighbourhood contacts is by definition spatially localized, trade movements could transmit infection between farms separated by much longer distances.

The simulations showed that even herds introducing yearly very few animals could be reached by the infection, in agreement with field observations in France [39]. The movement rate in the metapopulation together with the between-herd neighbourhood contacts were high enough in the studied situation to sustain infection persistence at a regional scale thanks to regular reintroductions of the virus at local scale, in agreement with previous results obtained using a theoretical framework [52]. A downward trend in the number of infected herds was predicted (Figure 4), which could be explained by a saturation effect occurring in endemic situations: once herd prevalence has reached quite a high level (predicted to be larger than 50% 3 years after the starting point of the simulation period), herd neighbourhoods become highly seroprevalent (i.e. immune), inducing a decrease in herd incidence through neighbourhood contacts. The role of trade movements as a transmission route does not seem to be much affected by a saturation effect, the number of newly infected herds due to this route being constant over time.

Trade movements have been recognized as an important, if not the main, transmission route in managed metapopulations, spreading several pathogens responsible for infectious diseases such as Johne’s disease [53], tuberculosis [14], Verotoxigenic *Escherichia coli* O157:H7 [54], and foot-and-mouth disease [55]. Although animal movements cannot necessarily maintain an infection at the regional scale without the implication of complementary transmission routes (e.g. vectorial transmission in vector-borne diseases such as bluetongue [15, 56]), it still plays an pivotal role in establishing new infections (as highlighted in [57]). Other transmission routes such as airborne transmission were shown to be important to explain the regional herd incidence in areas with a high cattle density and high prevalence (e.g. [58] for Q fever). As for BVDV, the contribution of neighbourhood contacts was highlighted by evaluating the risk of a dairy herd changing its status from absence to presence of P animals in relation with location and infection status of neighbouring herds [35]. However, this study did not allow assessing the relative roles of trade and neighbourhood contacts since movements were not included. Trade movements were the main contributor in transporting BVDV thereby influencing persistence and prevalence in infected herds and the size of epidemics in [39]. Neighbourhood contacts were negligible when assuming a random network in a very small metapopulation of one hundred herds. This conclusion does not to hold anymore when using a data-driven trade network of a much larger size in a densely populated region with cattle, highlighting the necessity to make use of realistic epidemiological models for achieving a better understanding of complex pathosystems.

Unsurprisingly, the number of neighbours and the number of purchased animals [59] appeared to be among the most important drivers of the probability to acquire BVDV infection through neighbourhood contacts and trade movements, respectively. More interestingly, the number of young animals in the herd and the number of young animals purchased by neighbouring herds also influenced the probability of new infection (Figure 6). Indeed, young animals are more vulnerable to infection since they are not yet immune. They are also an important source of potential infection, since highly contagious persistently infected animals are mostly calves (as P animals have a half-life period of 1 year [48]). This finding also illustrates the interplay between the two transmission routes: the infection can arrive by trade in the neighbourhood and then locally propagates by proximity contacts.

The proposed model includes all of the movements occurring between farms, but we neglected the intermediary stays in markets and assembly centres, which indeed have been shown to be quite frequent in the French cattle trade system [45]. Nevertheless, since most of these stays are short enough (1 day in markets and ~3 days in assembly centres), the risk of infection can be expected to be low. Such intermediate stays may exhibit in rare occasion a high risk of infection and subsequently impact BVDV spread through associated trade movements, giving rise locally to more movements of transiently infected animals and of dams carrying a persistently infected foetus than assumed in our model. Nevertheless, more information is required to account for such infection events during transportation, notably about the pregnancy status of traded females, which was not available in our datasets.

Current approximation for neighbourhood contacts assumes that all persistently infected animals from a given neighbourhood have an influence on all herds in this neighbourhood. Such an assumption might lead to over-estimate the influence of neighbourhood contacts for some herds and under-estimate it for some others. However, a more accurate representation of neighbourhood contacts would require a precise description of land use for pasture purpose, which was not available for the whole Brittany and which might be difficult to collect. For instance, the geographical coordinates of a farm usually point to the location of the “registered” housing unit, which does not necessarily correspond to pasture locations. Indeed, the pastures of a farm could be anywhere within a few kilometres around the geolocation of the farm and are not necessarily contiguous. Precise boundaries might not be obtainable due to various difficulties including privacy concerns. Moreover, data on pasture use are not of sufficient precision, being aggregated over all livestock species [60]. In the absence of these data, a simple approximation was designed by using the geographical coordinates of French commune borders and by randomly distributing herds within the borders of the commune they belong to. This approximation was shown not to impair our conclusions, infection dynamics being very similar when compared to simulations obtained with real geo-coordinates of farms in Finistère (Additional file 1B, Figure S2). In addition, beef herds also use pastures and thus could have neighbourhood contacts with dairy herds. They represent around 15% of cattle herds in Brittany, which was assumed to be low enough to be neglected. Besides, beef herds sell very few animals to dairy herds [61].

The model accuracy might be improved once more information would be available, for instance through a longitudinal regional epidemiological follow-up, that could be used to better infer parameters such as between-neighbour transmission rate for P animals ($\beta_{nb}^{P}$). This parameter was found to be the most important input factor of the sensitivity analysis (Figure 3) performed on three dynamical model outputs, at least in the range of parameter values considered. Despite the lack of detailed epidemiological data, we nevertheless calibrated the model by comparing the different simulated scenarios to observed herd prevalence and incidence data. These data correspond to Finistère, a subregion of Brittany containing 21% of its dairy herds distributed over 20% of its area and with a very similar trade pattern (Additional file 1A). This calibration, although qualitative, allowed to set the most influential and uncertain parameters to values compatible with data, and highlighted that neighbourhood contacts should be quite intense and within-herd transmission higher than expected for predictions to be in line with observations. Therefore, although not designed as a predictive epidemiological model for BVDV, this calibration conferred to our model a proof of plausibility with respect to data and legitimated the subsequent analyses of simulated scenarios.

This model could further serve as a tool to assess control strategies. Between-herd transmission not only occurs through trade movements of persistently infected animals and contacts with such animals located in the neighbourhood, but also through trade movements of transiently infected animals and of immune dams carrying a persistently infected foetus, which are much harder to detect [62]. According to field observations in French dairy cattle farms, despite collective control schemes implementing control of P animal movements and before pasture season, combined with test-and-cull of P animals in detected infected herds, it took several years to give rise to a decrease in prevalence without reaching full eradication [26]. Interestingly, our simulations showed that some herds are more prone to be exposed to one route over the other. This implies that the major between-herd transmission route should be considered specifically for each herd when designing control strategies aiming at eradication. All these results also indicate that regional control strategies have to be assessed according to territorial specificities in terms of prevalence of infection, intensity of trade movements, as well as intensity of neighbourhood contacts (herd and animal densities, duration of pasture season, risk of contact for gestating females, etc.). Our model can be used to investigate BVDV regional spread and evaluate control strategies in diverse epidemiological situations as long as similar datasets are available. Designed under an object-oriented framework, this model could be transferred as a support decision tool for animal health managers to evaluate adapted control and eradication strategies.

## Additional file

**Additional file 1.** **Complementary information to the material and method section.** (A) Characteristics of the trade network. (B) Comparison of model outputs from simulations based on real vs. random herd coordinates. (C) Newborn health states. (D) Sensitivity analysis. (E) Probability of herds to be more exposed to one route than the other. (F) Descriptive sensitivity analysis and comparison with summarized epidemiological observed data. (G) Herd infection probabilities. (H) Explanation of the IncMSE in Random Forest analysis.

## Abbreviations

BVDV: bovine viral diarrhoea virus
FNCD: French national cattle database
Prob_being_inf: annual probability of a herd to be infected, i.e. to have at least one persistently (P) or transiently (T) infected animal over the year
Prob_getting_inf: annual probability of a herd of acquiring infection, i.e. have at least one P or T animal in the year while none the previous year
nNB: number of herds in the neighbourhood
InStrength: annual number of animals purchased
InStrength_Less1yr: annual number of animals younger than 1 year of age purchased by the herd
InDegree: annual number of source herds
InStrengthNB: annual number of animals purchased by neighbouring herds
InStrengthNB_less1yr: annual number of animals younger than 1 year of age purchased by neighbouring herds
%24_36: proportion of cows aged 24 to 36 months in the herd
SizeTot: herd size
Size1yr: number of females younger than 1 year of age
