# Supplementary material for: Neighbourhood contacts and trade movements drive the regional spread of bovine viral diarrhoea virus (BVDV)
Source: Vet Res. 2019 Apr 29;50:30. doi: 10.1186/s13567-019-0647-x (PMC6489178; doi:10.1186/s13567-019-0647-x)
Supplement: Supplementary file 1 — Additional file 1. Complementary information to the material and method section. (A) Characteristics of the trade network. (B) Comparison of model outputs from simulations based on real vs. random herd coordinates. (C) Newborn health states. (D) Sensitivity analysis. (E) Probability of herds to be more exposed to one route than the other. (F) Descriptive sensitivity analysis and comparison with summarized epidemiological observed data. (G) Herd infection probabilities. (H) Explanation of the IncMSE in Random Forest analysis. [file 13567_2019_647_MOESM1_ESM.docx]

**Additional file 1. Complementary information to the material and method section**

(A) Characteristics of the trade network

(B) Comparison of model outputs from simulations based on real vs. random herd coordinates

(C) Newborn health states

(D) Sensitivity analysis

(E) Probability of herds to be more exposed to one route than the other

(F) Descriptive sensitivity analysis and comparison with summarized epidemiological observed data

(G) Herd infection probabilities

(H) Explanation of the IncMSE in Random Forest analysis

(I) References specifically used in Additional file 1

**A. Characteristics of the trade network**

Distributions of basic network characteristics (in- and out-degrees, i.e., number of trade partners, and in- and out-strengths, i.e. number of traded animals) were similar for herds located in Brittany and for herds located in Finistère, a sub-region of Brittany (Fig. S1), over the time period considered for simulations and scenario analysis in the main text (2005-2013).


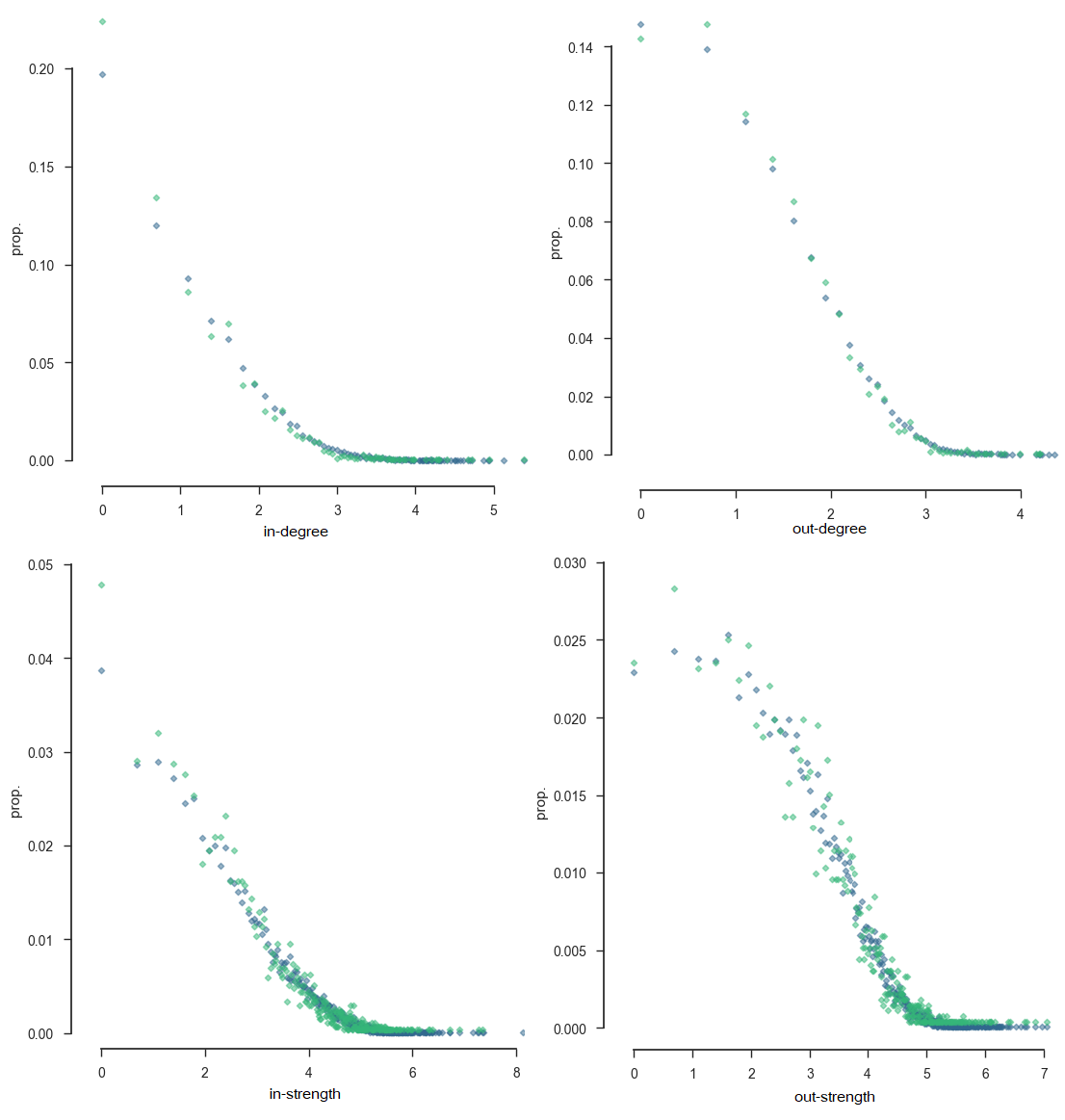


**Figure S1**. Characteristics of the cattle trade network for dairy herds located in Brittany (12,750 herds; blue dots) and in Finistère (2,663 herds; western part of Brittany; green dots), data aggregated over the period 2005-2013. Distributions of the in- and out-degrees (defined as the number of herds a given herd is connected to for purchasing and selling animals, respectively) and distributions of the in- and out-strengths (defined by the number of animals moved into and out of the herd, respectively).

**B. Comparison of model outputs from simulations based on real vs. random herd coordinates**

We compared model outputs generated over six years (2005-2010) for two scenarios: (1) when geographic coordinates of herds were randomly assigned within the known borders of the communes they belong to, and (2) real herd coordinates were used. Real coordinates were available for the 2,663 dairy cattle herds located in Finistère (western Brittany). Simulated trajectories were closely similar between the two scenarios for the four model outputs (Fig. S2).


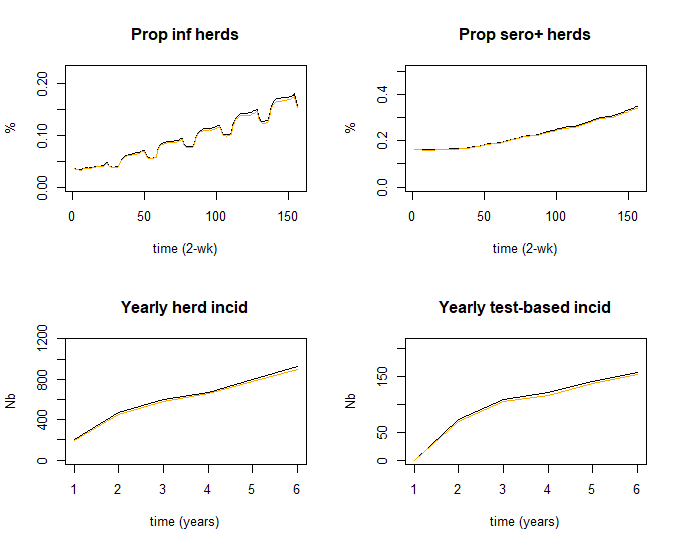


**Figure S2**. Comparison of model outputs for Finistère area over 2005-2010 when considering real herd geographic location (black) vs. randomly assigned locations for herds within the borders of communes they belong to (orange). Four model outputs were assessed: (i) the proportion of infected herds (which had at least one infected animals), top left; (ii) the proportion of herds with more than 10% of immune animals (i.e. animals of health status *R*), top right; (iii) the yearly herd incidence (number of herds which had at least one infected animals at time t while having none at time t-1, cumulated per year over all time steps), bottom left; and (iv) the yearly test-based herd incidence (herds that have been negatively tested twice during the previous year and twice positively during the current year, when using an ELISA test on collected milk, with a threshold of 10% of immune animals in the herd for test positivity), bottom right. Trajectories represent averages over 50 runs. Parameter values are: $\beta_{nb}^{P}$=0.20, *R_nb_*=4, $\beta_{w}^{T}$=0.06, $\beta_{b}^{P}$=0.20; see Tab. 1 in the main manuscript for the remaining parameters.

**C. Newborn health states**

At each time-step, the number of newborns in herd *y* was calculated using the estimated yearly birth rate for herd *y* and its simulated number of cows. The number of mothers was similar to the number of newborns. Health states of mothers were drawn from the simulated distribution of cows among health states in *herd y*. S, P, and T dams gave birth to S, P, and R calves, respectively. For R dams, random cows were chosen in the observed dataset. Their detention and trade histories, as well as past simulated herd structure containing the number of animals per age group per infection status (M, S, P, T, and R), were used to determine their most probable infection date according to infection statuses of herds they have belonged to before and during gestation (Fig. S3).


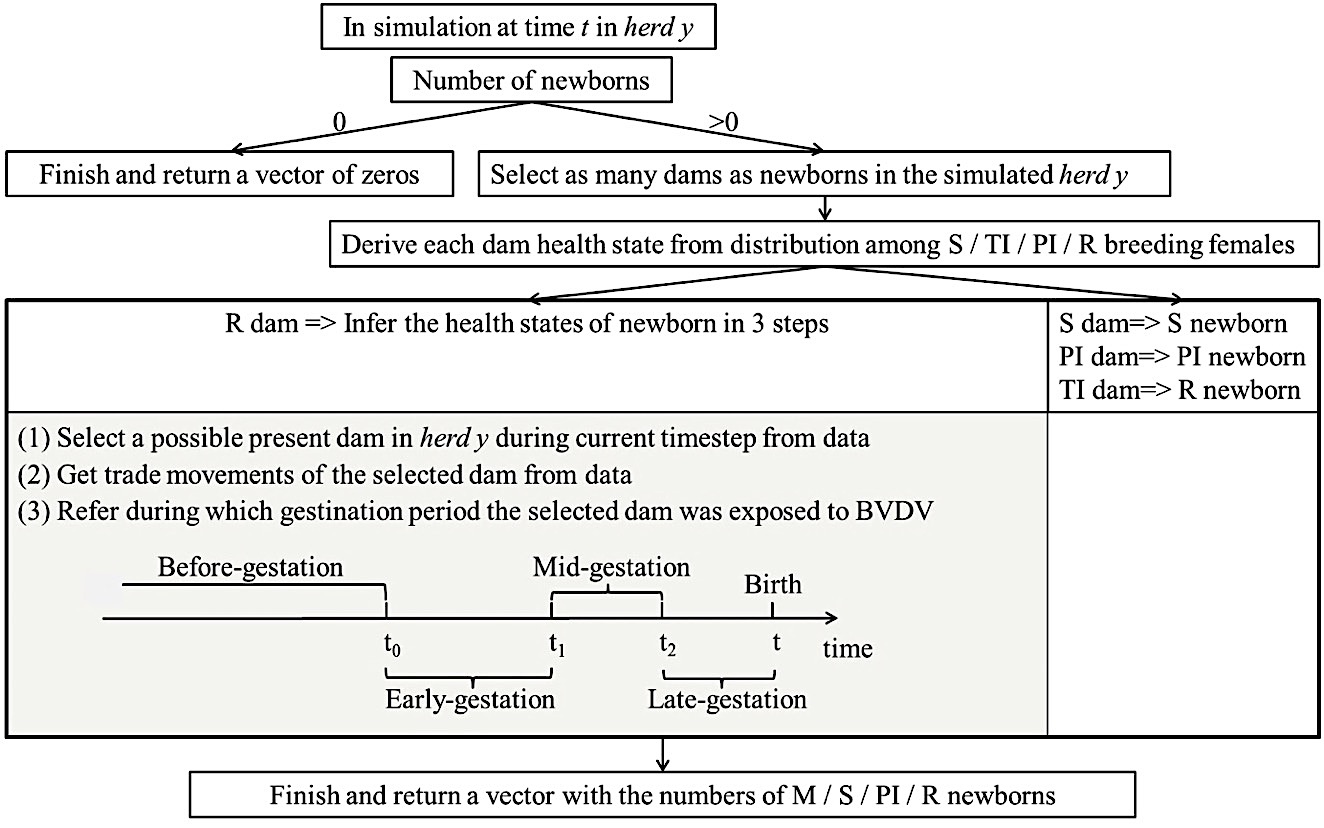


**Figure S3**. Proposed modelling procedure to determine calf health state at birth, with $t$ the current time, $t_{0}$ the start of gestation, $t_{1}$ and $t_{2}$ the range of mid-gestation during which vertical transmission might occur. Here PI and TI mean persistently and transiently infected animals, respectively.

At birth time *t*, the past was divided into three parts (Fig. S3): before mid-gestation (before *t*_1_), mid-gestation (between *t*_1_ and *t*_2_), and late-gestation (between *t*_2_ and *t*). Eighteen time intervals ($\Delta_{i}$, *i =* 0, 1, 2 … 17) were defined corresponding to eighteen probabilities ($r_{i}$). Time intervals $\Delta_{1}$ to $\Delta_{17}$corresponded to one time-step (2 weeks), while $\Delta_{0}$ represented the time before mid-gestation. Mid-gestation corresponded to $\Delta_{1},\cdots,\Delta_{8}$, and late-gestation to $\Delta_{9},\cdots,\Delta_{17}$. Each probability $r_{1},\cdots,r_{17}$ was calculated using the herd structure where the selected dam was at the corresponding time interval. $r_{0}$ represented the probability that the selected dam was not S at *t*_1_. As it was less precisely estimated than$r_{1},\cdots,r_{17}$, it was updated to 1 if $\sum_{i=1}^{i=17} r_{i}=0$. Probabilities $r_{0},r_{1},\cdots,r_{17}$ were initially set to zero. Assuming the selected dam arrived in *herd y* with health state *X* at time *T_x_* (*T_x_ < t*), five cases were considered:

1. If *X* = R or T and *T_x_ ≤ t*_1_, the newborn was M.
2. If *X* = R and *T_x_ > t*_1_, probabilities corresponding to [*t*_1_, *T_x_*] had to be updated.
3. If *X* = T, the dam was exposed during time interval $\Delta_{x}$, then $r_{x}=1$, $r_{i \neq x}=0$
4. If *X* = S and *T_x_ < t*_1_, probabilities $r_{0},r_{1},\cdots,r_{17}$ had to be updated.
5. If *X* = S and *T_x_ ≥ t*_1_, probabilities corresponding to [*T_x_*, *t*_2_] had to be updated.

Probabilities were updated using Eq. (S1):

$r_{0}=\left\{ \begin{matrix} 1,\sum_{i=1}^{i=17} r_{i}=0 \\ 1-\frac{S\left( t_{1} \right)}{S\left( t_{1} \right)+T\left( t_{1} \right)+R\left( t_{1} \right)} \end{matrix} \right. r_{i}=\left\{ \begin{matrix} 0,S\left( t_{i} \right)=0 \\ \frac{T\left( t_{i+1} \right)}{S\left( t_{i} \right)},S\left( t_{i} \right)>0 \end{matrix} \right. i=1,2,\ldots,17$ (S1)

with $S(t_{i})$, *T*(*t_i_*), and $R(t_{i})$ the number of animals in health states S, T, and R in the herd where the selected dam was located during $\Delta_{i}$, respectively. The period during which the selected dam was infected was randomly drawn in a multinomial distribution with probabilities $r_{0},r_{1},\cdots,r_{17}$. If in late-gestation, the newborn was R. If in mid-gestation, the newborn was P, M, or R with probabilities 0.934, 0.033, or 0.033 as assumed at the within-herd scale [1].

**D. Sensitivity analysis**

A sensitivity analysis was conducted to identify the parameters that mostly contributed to the output variability. Four critical transmission parameters (described in Tab. 1 of the main text; derived from [42]) were included in the sensitivity analysis as input factors: 2 of these parameters were assigned 2 values, and 2 other parameters were assigned 3 values (2*2*3*3=36 scenarios). Four other scenarios without neighbourhood contacts (only $\beta_{b}^{P}$ and $\beta_{w}^{T}$ were varied) were added, which makes a total of 40 scenarios. Each of these scenarios thus corresponded to a specific combination of parameter values. For each scenario, three outputs were simulated (defined in the main text, section 2.6), averaged over 50 runs.

To compare the influence of the input factors on the mean outputs exhibiting temporal dynamics, we applied a method developed by [R1] and implemented in the R package {multisensi}. For each output, an ANOVA was conducted at each time step, including the main effects and the two-factor interactions for all input factors (here the four model parameters). Sensitivity indices (SI), corresponding to the main effect, and global sensitivities (GSI), corresponding to the sum of the main effect and all the interactions, were calculated for each factor. In addition, a second analysis allowed to simultaneously consider all the time points for each output. A Principal Component Analysis (PCA) was operated in order to provide linear combinations (or components) of the initial variables (here the output values over time) explaining the maximum of inertia (i.e. variability) between scenarios. Only the first principal component was kept since it was sufficient to cover most variability among simulations. Then, a second ANOVA was performed on the first principal component and SI and GSI were calculated for each input factor.

**E. Probability of herds to be more exposed to one route than the other**

For each infected herd, we identified the causal infection route (neighbourhood contacts or trade movements) corresponding to the occurrence of the first within-herd infection event. Assuming the numbers of first within-herd infections caused by neighbourhood contacts (NB) and trade movements (MV) were *N* and *M* in all simulations, the probabilities of occurrence of each route ($p_{NB}^{Occur}$ and $p_{MV}^{Occur}$) in the metapopulation were obtained as follows:

$p_{NB}^{Occur}=\frac{N}{N+M}$ , $p_{MV}^{Occur}=\frac{M}{M+N}$ (S2)

Then, we collected all the within-herd infection events in the simulations and recorded the cause for each infection. For all the non-first within-herd infections, we found the corresponding previous infection in the same herd and recorded its route. If one infection was caused by neighbourhood contacts and its previous one was also caused by neighbourhood contacts, then a NB-NB pattern was identified and counted. Following the same procedure, a set of four possible patterns were identified: Ω = {NB-NB, NB-MV, MV-MV and MV-NB}. The probability of the occurrence of pattern *x* $(p_{pattern x}^{Observation}$) from observation was calculated as:

$$p_{pattern x}^{Obervation}=\frac{Nx}{\sum_{x\in\Omega} Nx} (S3)$$

where $Nx$ is the number of occurrences of pattern *x*, with $x\in\Omega.$

By assuming two successive within-herd infections are independent of the herd, we calculated the probability of the occurrence of each pattern ($p_{pattern x}^{Occur}$):

$p_{x-y}^{Occur}=p_{x}^{Occur}*p_{y}^{Occur}$ (S4)

By comparing results obtained from Eq. (S3) and Eq. (S4), the probability of herds to be more exposed to one route than the other can be deduced given the route of the previous infection.

**F. Descriptive sensitivity analysis and comparison with summarized epidemiological observed data**

From the descriptive sensitivity analyses, we retained the following values for the epidemiological parameters, after a visual inspection assessing the qualitative agreement with aggregated observed data (54-62% seroprevalence, and from 83 to 390 of herds yearly detected as newly infected): $\beta_{nb}^{P}$=0.20, *R_nb_*=4, $\beta_{w}^{T}$=0.06, $\beta_{b}^{P}$=0.20, other parameters being at their reference value (main text, Tab. 1). This corresponds in Fig. S5 and S6 to the orange curve of the sub-panel in the middle of the bottom line. Larger parameter values would have been unrealistic, leading to far too high within-herd prevalence of infection which has been observed to be around 1-2% of persistently infected animals per infected herd on average [24].


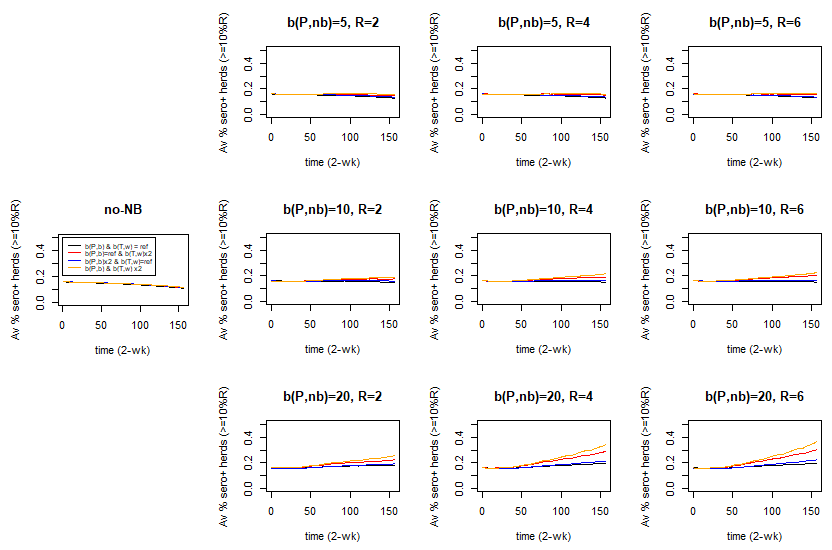


**Figure S5**. Sensitivity analysis. Variation of the proportion of seropositive herds (%R>10%) with respect to variation in epidemiological parameters defined here as: b(P,b) = $100* \beta_{b}^{P}$ the between-group within-herd transmission rate due to persistently infected (P) animals; b(T,w) = $\beta_{w}^{T}$ the within-group within-herd transmission rate due to transiently infected animals; b(P,nb) = $\beta_{nb}^{P}$ the between-herd transmission rate due to P animals located in the neighbourhood, R = *R_nb_* the radius of the neighbourhood (no-NB for no consideration of neighbourhood contacts). The variation of the first two parameters is represented by the four coloured curves in each panel. The variation of the two remaining parameters corresponds to the different panels. Reference values (ref) are provided in the main text (Tab. 1).


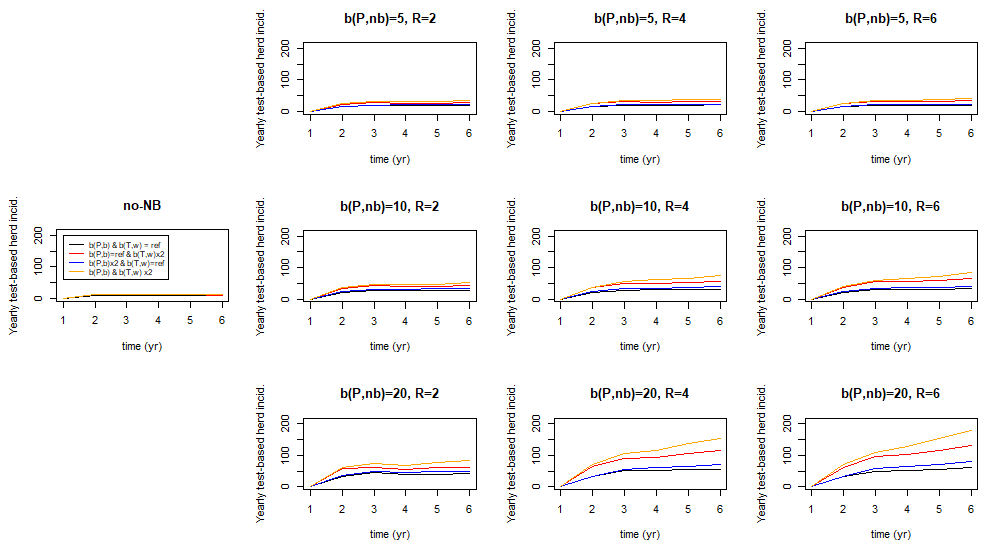


**Figure S6**. Sensitivity analysis. Variation of the yearly test-based herd incidence (defined in main text) with respect to variation in epidemiological parameters defined here as: b(P,b) = $100* \beta_{b}^{P}$ the between-group within-herd transmission rate due to persistently infected (P) animals, b(T,w) = $\beta_{w}^{T}$ the within-group within-herd transmission rate due to transiently infected animals, b(P,nb) = $\beta_{nb}^{P}$ the between-herd transmission rate due to P animals located in the neighbourhood, R = *R_nb_* the radius of the neighbourhood (no-NB for no consideration of neighbourhood contacts). The variation of the first two parameters is represented by the four coloured curves in each panel. The variation of the two remaining parameters corresponds to the different panels. Reference values (ref) are provided in the main text (Tab. 1).


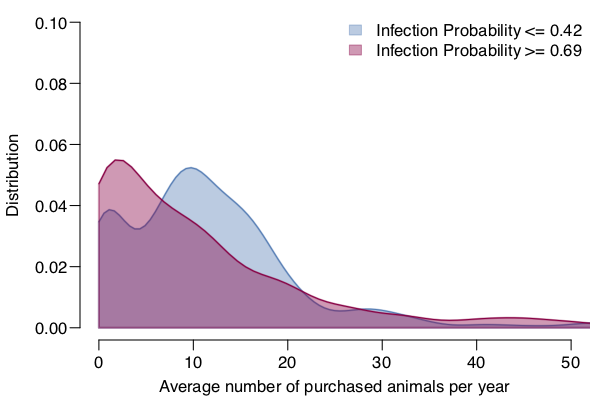
**
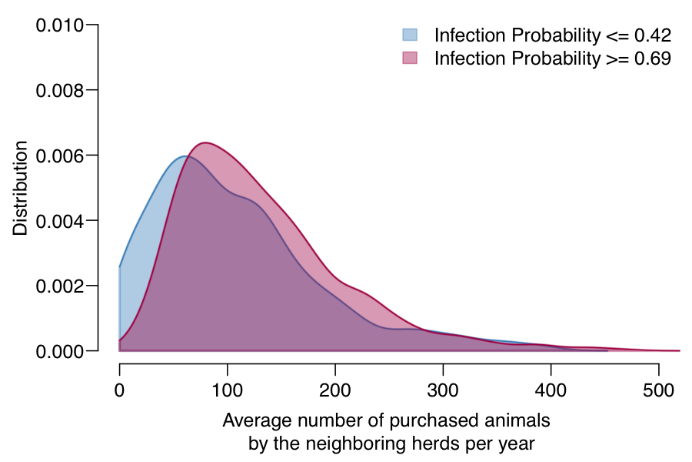
**
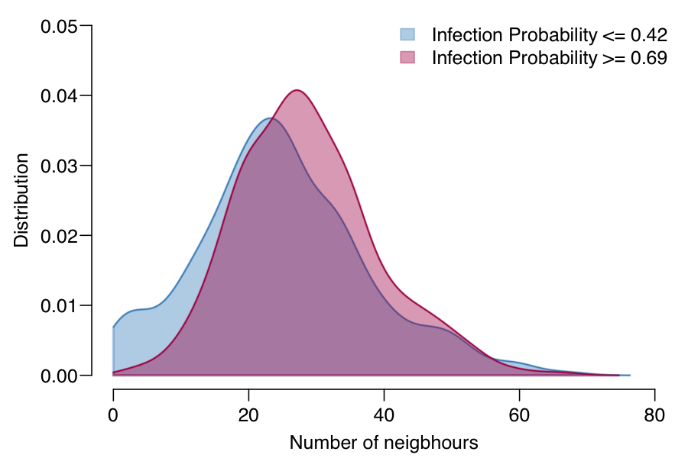
**G. Herd infection probabilities**

**Figure S7.** Distributions of the number of neighbours (top left; significantly different using a t-test for mean comparison, p-value<10^-7^), of the average number of purchased animals per year by neighbouring herds (top right; significantly different, p-value <10^-7^), and of the average number of purchased animals per year (bottom left; not significantly different, p-value = 0.73) for herds in extreme percentiles (5 in blue, and 95 in pink) of infection probability (*Prob_tobeinf_byP*).

**H. Explanation of the IncMSE in Random Forest analysis**

The following text comes from the tutorial on the R package {randomForest}, corresponding to variable importance. “IncMSE is computed from permuting out-of-bag (OOB) data. For each tree, the prediction error on the OOB data is recorded (error rate for classification, MSE for regression). Then, the same is done after permuting each predictor variable. The difference between the two are averaged over all trees, and normalized by the standard deviation of the differences. If the standard deviation of the differences is equal to 0 for a variable, the division is not done (but the average is almost always equal to 0 in that case).”

**I. References specifically used in Additional file 1**

R1. Lamboni, M., Monod, H., Makowski, D., 2011. Multivariate sensitivity analysis to measure global contribution of input factors in dynamic models. Reliability Engineering & System Safety, 96:450-459, <https://doi.org/10.1016/j.ress.2010.12.002>.

Other reference numbers refer to the list provided in main text.
